# DNAMarkMaker: streamlining ARMS and CAPS marker development from resequencing data with NGS short reads

**DOI:** 10.1270/jsbbs.23048

**Published:** 2024-02-29

**Authors:** Tenta Segawa, Sorachi Saiga, Marina Takata, Riki Kumazawa, Makishi Hara, Hiromoto Yamakawa, Hiroki Takagi

**Affiliations:** 1 Ishikawa Prefectural University, 1-308 Suematsu, Nonoichi, Ishikawa 921-8836, Japan; 2 Institute of Crop Science, National Agriculture and Food Research Organization (NARO), 2-1-2 Kannondai, Tsukuba, Ibaraki 305-8518, Japan

**Keywords:** DNA marker, ARMS, CAPS, Next-generation sequencing, SNP

## Abstract

DNA markers serve as essential tools in breeding selection and genetic analysis. However, developing DNA markers can be time-consuming and labor-intensive due to the need to identify polymorphisms between cultivars/lines and to design suitable primers. To address these challenges, we have developed DNAMarkMaker, a tool designed to automate the process of primer design for Amplification Refractory Mutation System (ARMS) and Cleaved Amplified Polymorphic Sequences (CAPS) markers, utilizing resequencing data. One key feature of DNAMarkMaker is its user-friendly graphical user interface (GUI), ensuring its accessibility and ease of use, even for researchers not well-versed in bioinformatics. We confirmed DNAMarkMaker’s applicability by developing DNA markers for rice, potato, and turnip—each representing distinct genome structures: homozygous diploid, heterozygous autotetraploid, and heterozygous diploid, respectively. DNAMarkMaker will contribute to the rapid and efficient development of DNA markers, accelerating breeding and genetic analysis in various crops.

## Introduction

DNA markers play a pivotal role in plant breeding, serving functions ranging from the selection of individuals with desired alleles to facilitating genetic analyses, verifying crossbreeding events, and distinguishing specific cultivars or lines (Collard and Mackill 2008). In modern crop breeding, DNA marker-assisted selection (MAS) has been one of the most effective strategies. MAS, which utilizes DNA markers closely linked to genes controlling target traits, circumvents the labor-intensive and time-consuming process of phenotyping (Hasan *et al.* 2021).

Among various types of DNA markers, such as Restriction Fragment Length Polymorphism (RFLP), Amplified Fragment Length Polymorphism (AFLP), and Random Amplification of Polymorphic DNA (RAPD) (Blears *et al.* 1998, Botstein *et al.* 1980, Williams *et al.* 1990) have been predominantly applied when sequence information containing the polymorphisms is unavailable. However, the efficacy of these markers in detecting polymorphisms can differ significantly based on the chosen sample, targeted genomic region, and specific experimental conditions. As a result, marker development often required repeated experimentation to successfully detect a polymorphism, making it a labor-intensive and time-consuming effort. Advances in sequencing technology, including Sanger sequencing and bacterial artificial chromosome (BAC) clones (Han and Xue 2003), have enabled the detection of sequence-level polymorphisms. Consequently, more accurate and efficient genotyping can be achieved by designing primers at the flanking regions of these polymorphisms. Simple Sequence Repeat (SSR) markers, which detect polymorphisms based on variations in the number of SSR, have been widely used both in genetic analysis and in MAS during breeding (Powell *et al.* 1996) because the SSR markers are applicable to various cultivars exhibiting polymorphisms by simple PCR experiments with a primer pair designed at the flanking region. However, SSR markers often lack polymorphism when comparing genetically close cultivars, making their application potentially ineffective. Therefore, single nucleotide polymorphisms (SNPs) have been targeted for marker development due to their abundance in the genome even among genetically similar cultivars.

Among the SNP-targeted markers, Amplification Refractory Mutation System (ARMS) and Cleaved Amplified Polymorphic Sequences (CAPS) markers have been frequently used in recent study and breeding efforts (Konieczny and Ausubel 1993, Newton *et al.* 1989). The ARMS marker enables genotyping through the amplification of an allele-specific PCR product using primers located at the position showing polymorphisms at the 3ʹ end. The CAPS marker enables genotyping based on differences in PCR products digested by a specific restriction enzyme, which recognizes and cleaves at the polymorphic sites. Both ARMS and CAPS markers allow the precise and rapid genotyping even in closely related cultivars/lines. However, the prerequisite of identifying polymorphisms to design primers for ARMS and CAPS markers presents a challenge. This requirement has limited the application of ARMS and CAPS markers.

Advances in Next-generation sequencing (NGS) have significantly reduced both time and cost associated with sequencing (Hu *et al.* 2021). Furthermore, advancements in NGS-based genome assembly have facilitated an increase in the availability of public reference genomes across various plant species (Sun *et al.* 2022). As a result, resequencing techniques have become available for a wide range of crops, allowing for easy identification of SNP positions across whole genome (Kumar *et al.* 2012). However, developing ARMS and CAPS markers demands substantial effort for designing primers that account for mismatches at the 3ʹ end and identifying the SNP within the sequence recognized by a restriction enzyme. The public tools, such as SNP2CAPS and PRIMER1, are available for assistance in designing CAPS and ARMS markers, respectively (Collins and Ke 2012, Thiel *et al.* 2004). However, these tools are restricted to sequences of limited length in FASTA format files. This suggests that the vast number of SNP positions identified through resequencing cannot be applied to marker development. Consequently, there is a great demand for novel tools capable of designing primers for DNA markers that target SNPs identified through resequencing.

In this study, we developed DNAMarkMaker, a tool designed to streamline the development of ARMS and CAPS markers for a given genomic region from resequencing result. To verify the applicability of DNAMarkMaker, we applied it to a range of plants including rice with a homozygous diploid genome, potato with a heterozygous autotetraploid genome and turnip with a heterozygous diploid genome.

## Materials and Methods

### Whole genome sequencing

DNA was extracted from rice (*Oryza sativa*) cultivars ‘Ishikawa 65’ and ‘Ishikawa Sake 68’ using the DNeasy Plant Mini Kit (QIAGEN), followed by library preparation for Illumina sequencing platform with the NEBnext Ultra II FS DNA Library Prep Kit (NEB). These prepared libraries were then sequenced using Illumina HiSeqX (Illumina). The NGS reads for turnip (*Brassica rapa*) and potato (*Solanum tuberosum*) were downloaded from the NCBI Sequence Read Archive (SRA) using SRA-tools (https://github.com/ncbi/sra-tools.git). The accession number for each NGS read was summarized in Supplemental Table 1.

In resequencing, public reference sequences ‘IRGSP-1.0’ for rice (https://rapdb.dna.affrc.go.jp), ‘DM 1-3 516 R44 v6.1’ for potato (http://spuddb.uga.edu/index.shtml) and ‘Chiifu V4.0’ for turnip (http://brassicadb.cn/#/) were used without the scaffold sequences (Pham *et al.* 2020, Sasaki and Burr 2000, Wang *et al.* 2011). The alignment of NGS reads to the reference sequence was conducted by BWA ver 0.7.17 (Li and Durbin 2009). The alignment data was then converted from SAM to BAM formatted file, and PCR duplications were removed using Samtools ver 1.16.1 (Li *et al.* 2009).

### Configuration of DNAMarkMaker

Both GUI and CUI type DNAMarkMaker are distributed in GitHub (https://github.com/SegawaTenta/DNAMarkMaker-GUI and https://github.com/SegawaTenta/DNAMarkMaker-CUI). GUI DNAMarkMaker works in macOS. CUI DNAMarkMaker works in macOS and Linux.

In DNAMarkMaker, users can define conditions for selecting SNP positions and designing primers as described in the results section. The condition applied to each crop was summarized in Supplemental Table 2. The default configuration for designing primers in this study was also summarized in DNAMarkMaker manual (https://github.com/SegawaTenta/DNAMarkMaker_manual) as recommended values. The simulation datasets utilized for defining the heterozygous genotype can be accessed at the same URL as provided for the manual. DNA markers were developed for each crop throughout the genome at 1 Mb intervals.

### PCR conditions

For all ARMS and CAPS assays in this study, PCR amplifications were conducted using rTaq (TOYOBO). The conditions were set to initiate at 94°C for 1 min, followed by 30 cycles (98°C for 10 sec, 60°C for 30 sec, 72°C for 60 sec), and a final extension at 72°C for 7 min. The ProFlex^TM^ PCR System (ThermoFisher) was utilized as the thermal cycler for all PCR reactions in this study.

## Results

### The workflow of DNAMarkMaker

**Fig. 1.**
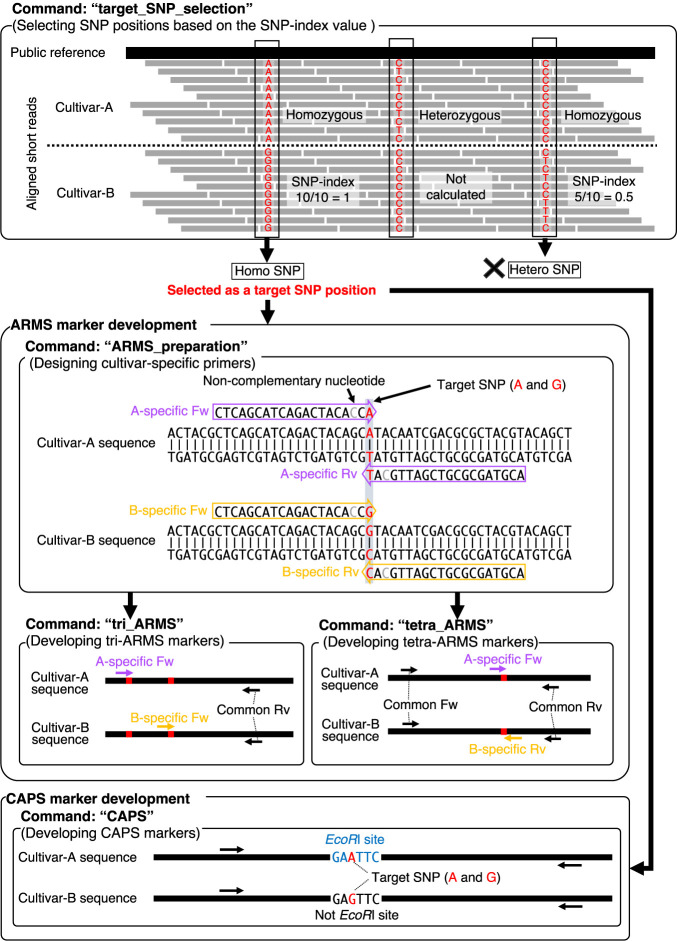
Flowchart of DNAMarkMaker operations. This diagram describes the process flow for using DNAMarkMaker to develop markers at homozygous SNP positions between cultivar-A and -B.

DNAMarkMaker is software designed to automatically generate ARMS and CAPS markers at SNP sites, identified through the resequencing of two cultivars. This section outlines the two-step DNAMarkMaker workflow: 1) SNP position selection, and 2) primer design, specifically for the development of markers between cultivars-A and -B (Fig. 1).

The SNP position selection step identifies SNP positions suitable for generating marker from alignment files resulted from resequencing cultivar-A and -B against a publicly available reference genome. The identified SNP positions are specific to cultivar-B at locations where cultivar-A does not display any polymorphisms. From these cultivar-B specific SNP positions, those exhibiting the target genotype are selected, based on the SNP-index value. This SNP-index value represents the frequency of NGS reads containing the cultivar-B specific allele among the total number of NGS reads covering that position (Abe *et al.* 2012, Itoh *et al.* 2019). The final set of selected SNP positions are then utilized for designing primers to develop ARMS and/or CAPS markers.

In the development of ARMS markers, the primer design step initiates with the design of cultivar-specific primers that target selected SNPs. Following this, the common primer paired to these cultivar-specific ones are designed, based on consensus sequences shared between the two cultivars. The Primer3 software (Untergasser *et al.* 2012) is applied to design each primer. The design of the cultivar-specific primer ensures that its 3ʹ terminal nucleotide aligns with the selected SNP position. In addition, the nucleotide located 2 bases upstream from the 3ʹ terminal nucleotide is substituted with a non-complementary nucleotide for increasing the specificity in annealing stage of PCR (Medrano and Oliveira 2014, Ye *et al.* 2001). During the primer design, it is ensured that Primer3 parameter criteria, including GC content, Tm value, and length, are satisfied. It’s also verified that the consensus region, from which the common primer is designed, contains no mutations between the cultivars. At all selected SNP positions, both forward and reverse cultivar-specific primers for cultivar-A and -B nucleotide are designed, potentially resulting in the design of up to four primers at a SNP position. The common primers for both cultivars are designed according to the number and orientation of the cultivar-specific primers at each SNP position, for potential use as tri-ARMS or tetra-ARMS markers. If both cultivar-A and -B specific primers exist in the same orientation within a defined distance, a common primer of the opposite orientation is designed, resulting in a tri-ARMS marker. On the other hand, if cultivar-A and -B specific primers are present in opposite orientations at a SNP position, a pair of common primers is designed at the flanking region of the SNP position, resulting in a tetra-ARMS marker.

CAPS marker development involves verifying the existence of restriction enzyme sites at the selected SNP positions and subsequently designing primer pairs at flanking region of these restriction enzyme sites. Similar to ARMS markers, the designed primers are validated to satisfy the Primer3 parameter conditions and show no SNPs within the primer region.

### How to use DNAMarkMaker and interpret its results

**Fig. 2.**
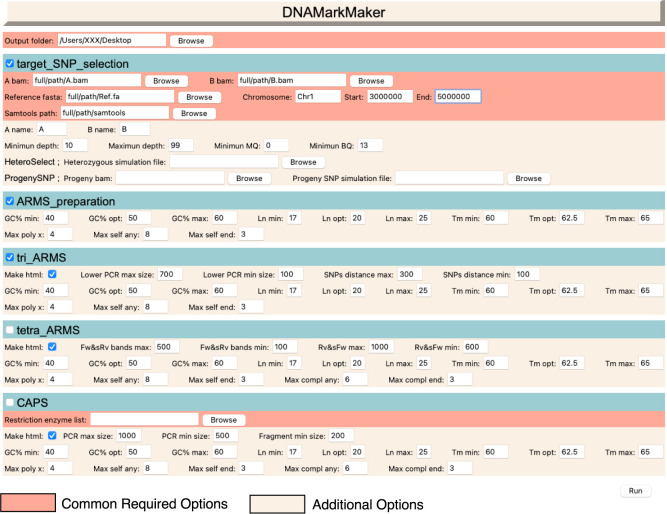
Input screen for DNAMarkMaker. This figure illustrates the input screen of the DNAMarkMaker tool when users design tri-ARMS marker in cultivar-A and -B.

DNAMarkMaker is a user-friendly graphical user interface (GUI) designed for biologists who are unfamiliar with command-line operations. Users can develop DNA markers using the alignment files (BAM format) for two cultivars and their reference file (FASTA format). The general workflow for using DNAMarkMaker is outlined below (Fig. 2).

DNAMarkMaker incorporates five execution commands: “target_SNP_selection”, “ARMS_preparation”, “tri_ARMS”, “tetra_ARMS” and “CAPS”. During the development of ARMS markers, the process initiates with the “target_SNP_selection” command to select the target SNP positions within a genomic region specified by the user. This is followed by the “ARMS_preparation” command, which designs cultivar-specific primers at the selected SNP positions. Finally, either the “tri_ARMS” or “tetra_ARMS” command is employed to design common primer and develop each type of ARMS markers from primers that meet the design criteria.

For CAPS marker development, the process is similar to that of ARMS markers. It initiated with the “target_SNP_selection” command to select the appropriate SNP positions. Then, the “CAPS” command with restriction enzyme list is used to develop CAPS markers by designing primers for the flanking regions containing restriction enzymes site listed by user.

**Fig. 3.**
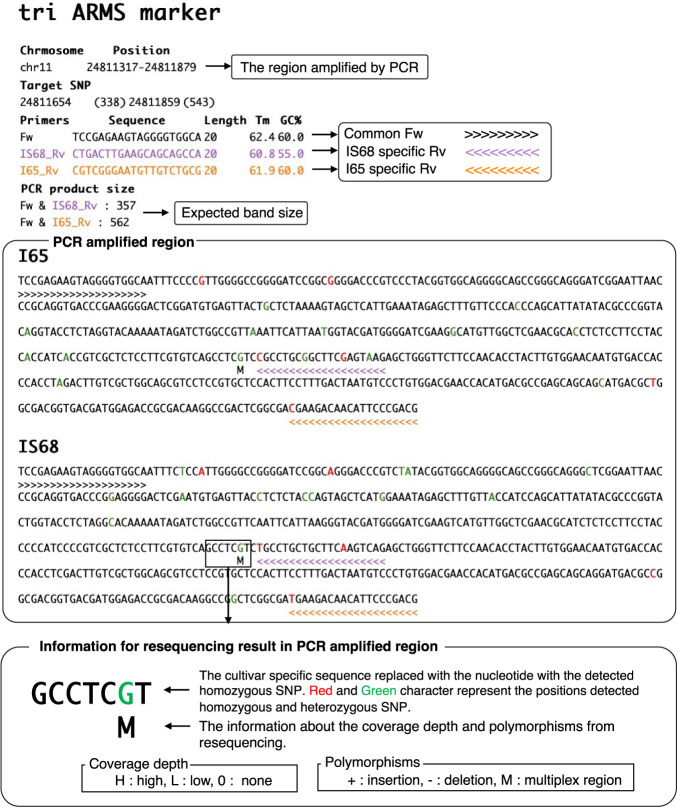
DNAMarkMaker output. This figure presents an interpreted HTML file, viewable in any internet browser, displaying a tri-ARMS marker as output from DNAMarkMaker.

Both ARMS and CAPS markers developed with DNAMarkMaker are outputted in an HTML file which can be easily viewed in an internet browser (Fig. 3, Supplemental Fig. 1). This HTML file provides information such as the region amplified by each marker, product size, the target SNP positions, and the primer sequence. Notably, positions that display polymorphisms or those that lie outside a pre-defined coverage depth range within the PCR product are discerned from the resequencing data and accentuated in distinct colors. This comprehensive information allows users to select the most appropriate marker for clear distinction of genotype. If the markers are designed at the region having less coverage depth and many polymorphisms, users should avoid such markers (Supplemental Fig. 1).

### Development of DNA markers in highly homozygous plant species

**Fig. 4.**
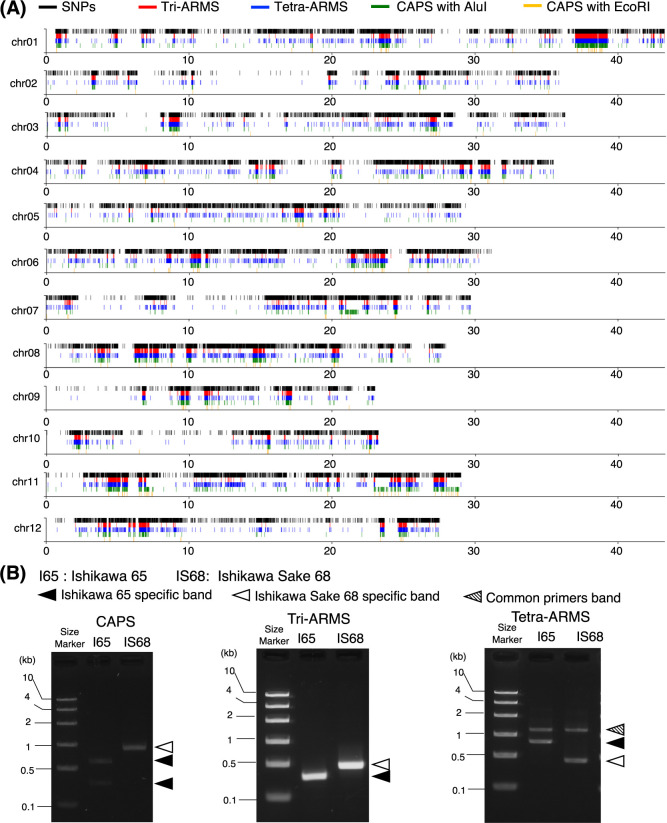
Utilizing DNAMarkMaker for rice—an example of a homozygous diploid plant. (A) The positions of SNPs and the developed markers between rice cultivars ‘Ishikawa 65’ and ‘Ishikawa Sake 68’. This panel illustrates the distribution of SNP positions and each generated marker, with each denoted by a different colored line. (B) Electrophoresis images of the PCR products obtained using CAPS, tri-ARMS, and tetra-ARMS markers developed by DNAMarkMaker. A schematic diagram of the designed primers is shown in Supplemental Fig. 2.

To validate the utility of DNAMarkMaker for developing ARMS and CAPS markers in plants with highly homozygous genomes, we applied DNAMarkMaker to two rice cultivars: ‘Ishikawa 65’, an eating cultivar, and ‘Ishikawa Sake 68’, a sake brewing cultivar, both developed by Ishikawa Prefecture. Initially, using “target_SNP_selection” with default configuration, a total of 149,614 homozygous SNP positions exhibiting SNP-index = 1 in ‘Ishikawa Sake 68’ against to ‘Ishikawa 65’ were selected from resequencing data of ‘Ishikawa 65’ and ‘Ishikawa Sake 68’ on the Nipponbare reference genome ‘IRGSP-1.0’ (Fig. 4A). Then, “ARMS_preparation” designed 74,461 and 71,694 primers specific to ‘Ishikawa 65’ and ‘Ishikawa Sake 68’, respectively, at the identified 149,614 homozygous SNP positions. Finally, “tri_ARMS” generated 98,765 tri-ARMS markers by combining dual cultivar-specific primers and the common primers. The dual cultivar-specific primers were designed within the range from 100 to 300 bp, while common primers were designed within a range from 100 to 700 bp from one of the designed cultivar-specific primers. In related processes, the “tetra_ARMS” command developed 15,708 tetra-ARMS markers across the whole genomic region, accounting for 10.49% of all identified homozygous SNP positions. On the other hand, “CAPS” command developed 2,826 CAPS markers using AluI (a 4-bp cutter) and 195 CAPS markers using EcoRI (a 6-bp cutter) at 149,614 positions selected by “target_SNP_selection”. These results suggest that 1.88% and 0.13% of all identified homozygous SNP positions were utilized for the development of CAPS markers with these 4 and 6 bp cutters, respectively.

We validated the function of one marker each from the developed tri-ARMS, tetra-ARMS, and CAPS categories for genotyping (Fig. 4B, Supplemental Fig. 2). The results for each marker showed the expected band pattern, indicating that DNAMarkMaker enables the efficient development of DNA markers in homozygous plants.

### Selecting SNP positions for DNAMarkMaker to target heterozygous positions

The “HeteroSelect” option within the command “target_SNP_selection” allows for the selection of heterozygous SNP positions with the simulation data of SNP-index values (Supplemental Fig. 3). The simulated SNP-index values depend on the coverage depth of resequencing and the genome structure of the samples. Because heterozygous SNP positions in diploid plants have two different alleles, the expected SNP-index values at these positions should be close to 0.5, given the equal ratio of NGS reads containing each allele. On the other hand, for heterozygous SNP positions in polyploid plants, variant allele dosage must be considered. For instance, heterozygous genotypes in autotetraploid species, such as potatoes, are classified into three types: simplex, duplex and triplex, that have one, two and three variant alleles, respectively. For developing markers that target the simplex genotype in autotetraploid plants, the SNP-index values at the SNP position selected by “HeteroSelect” should be close to 0.25 because only one out of four alleles carries a variant allele at the SNP position. The “HeteroSelect” option enables users to select heterozygous SNPs suitable for marker development based on the confidence interval of the SNP-index value from simulation datasets reflecting the sample’s ploidy and variant allele dosage.

**Fig. 5.**
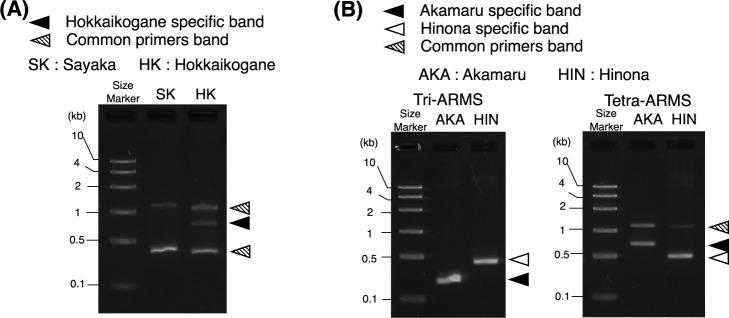
Applicability of DNAMarkMaker for a heterozygous autotetraploid and heterozygous diploid plant. Electrophoresis images of the PCR products obtained using a tetra-ARMS marker in potato (A) and both tri-ARMS and tetra-ARMS markers in turnip (B). A schematic diagram of the designed primers is shown in Supplemental Fig. 2.

To demonstrate the applicability of “HeteroSelect”, we developed ARMS markers at a simplex type of heterozygous position between the autotetraploid potato cultivars ‘Sayaka’ and ‘Hokkaikogane’. Although heterozygous SNPs with an SNP-index >0 and <1 were detected at 11,556,508 positions, “HeteroSelect” with simplex simulation data selected 2,248,618 of these positions as candidate positions for developing a DNA marker between ‘Sayaka’ and ‘Hokkaikogane’. Subsequently, “ARMS_preparation” designed 465,814 primers, and “tetra_ARMS” developed 11,100 tetra-ARMS markers. Using one of these 11,100 markers, we conducted a PCR test and validated the expected PCR products (Fig. 5A, Supplemental Fig. 2). The developed tetra-ARMS marker successfully confirmed the positions that possessed a single dosage of the ‘Hokkaikogane’-specific allele.

### Increasing accuracy of the selected SNP positions in the plants with highly heterozygous diploid genome

Among practically important crops, there are plant species with highly heterozygous diploid genomes. For example, many plant species belonging to the Brassicaceae family exhibit self-incompatibility, a trait that contributes to the heterozygosity of their genomes. Consequently, even local landraces, known for their phenotypically stable lines/cultivars, may possess a highly heterozygous genome. If the individual sequenced for use with DNAMarkMaker is different from the individual used for crossing to generate segregating progeny and breeding lines, there is a possibility that the developed DNA marker does not function.

To develop a DNA marker that consistently functions across individuals within lines/cultivars, the “ProgenySNP” option within the “target_SNP_selection” command can be employed. This option selects SNP positions that display a heterozygous state in the progeny resulting from a cross between samples used with DNAMarkMaker (Supplemental Fig. 4).

Here, we used “ProgenySNP” for developing ARMS markers between turnip cultivars ‘Akamaru’ and ‘Hinona’. For the resequencing data from the progeny, we used sequence data derived from randomly bulked F_2_ individuals of ‘Akamaru’ and ‘Hinona’ both of which are different from the individuals sequenced for DNAMarkMaker. The DNA from these randomly bulked F_2_ individuals is expected to have an equal ratio of ‘Akamaru’ and ‘Hinona’ alleles at all SNP positions. This corresponds to the DNA from the F_1_ generation, which exhibits a heterozygous state at all SNP positions. While the default setting of “target_SNP_selection” initially pinpointed 326,242 homozygous SNP positions between ‘Akamaru’ and ‘Hinona’, utilizing the “ProgenySNP” option to select positions displaying a heterozygous state in the F_2_ random bulk reduced this number to 190,943 SNP positions suitable for DNA marker development. These selected positions likely represent SNP present in both the sequenced and crossed individuals. Following the SNP position selection, “ARMS_preparation” followed by “tri_ARMS” and “tetra_ARMS” primers developed 8,176 and 755 markers, respectively. The developed markers produced the expected PCR products, thus confirming their function (Fig. 5B, Supplemental Fig. 2).

### The relation between SNP density and type of ARMS marker

**Fig. 6.**
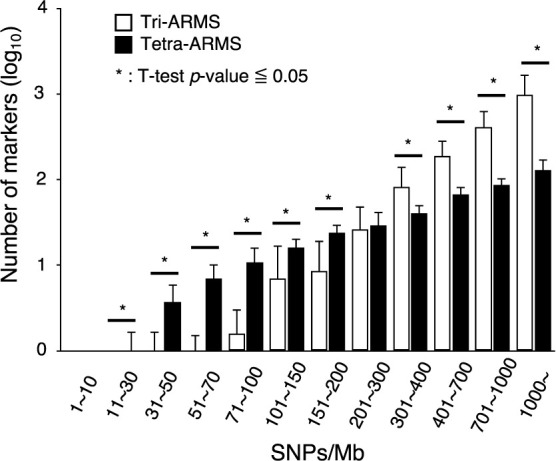
Relationship between SNP density and the number of developed ARMS marker types. This bar graph shows the number of tri-ARMS and tetra-ARMS markers developed in genomic regions with varying SNP densities in the rice sample described in Fig. 4.

DNAMarkMaker is capable of developing two types of ARMS markers: tri-ARMS and tetra-ARMS. To estimate the suitable SNP density for designing tri-ARMS or tetra-ARMS marker, we compared the number of tri-ARMS and tetra-ARMS markers developed in different SNP density using the rice example described in the previous section (Fig. 6).

In regions with a SNP density of less than 150 SNPs/Mb, few tri-ARMS markers were designed, while an average of 6.3 tetra-ARMS markers were generated. In cases that the SNP density exceeded 150 SNPs/Mb, the number of tri-ARMS markers begins to rise although until the SNP density reaches 200 SNPs/Mb, more tetra-ARMS markers are developed. When the SNP density exceeds 300 SNPs/Mb, the number of tri-ARMS markers became higher than tetra-ARMS markers.

For the rice sample in this study, genomic regions with a SNP density of 1–200 SNPs/Mb covered 74.09% of the total genomic area (Supplemental Fig. 5). Hence, in this rice sample, the development of the tetra-ARMS marker would likely be more appropriate for genotyping across a broader genomic region.

## Discussion

In this study, we have developed DNAMarkMaker, a GUI application that extracts SNPs from resequencing data and utilizes Primer3 to design primers for both ARMS and CAPS markers.

DNAMarkMaker enables application to various plants, not only to rice with a homozygous diploid genome, but also to turnip with a highly heterozygous diploid genome, and to potatoes with a highly heterozygous autotetraploid genome. This versatility is achieved using the filter options in “target_SNP_selection”, which allow users to select SNP positions suitable for DNA marker development. However, developing DNA markers in allopolyploid plants isn’t straightforward due to their homologous regions between subgenomes; amplification from non-targeted subgenomes must be avoided. Saiga *et al.* (2023) successfully developed a DNA marker for strawberry, an allooctaploid plant, by ensuring the primer sequence was absent in non-targeted subgenomes through inter-subgenome sequence comparison. When using DNAMarkMaker for allopolyploid plants, it is essential to verify the specificity of the primers designed for the targeted subgenome through tools like BLAST or subgenome alignment.

DNAMarkMaker allows users to choose the appropriate ARMS marker type based on the characteristics of the tri- and tetra-ARMS markers and the genome structure of the samples. A correlation between SNP density and the number of markers suggests that designing tri-ARMS markers is facilitated in genomic regions exhibiting high density of SNP positions. Moreover, compared with tetra-ARMS markers, tri-ARMS markers reduced the risk of primer dimer and non-specific amplification by reducing the number of primers. In contrast, tetra-ARMS markers can be designed in broader genomic regions without consideration of SNP density. Moreover, the tetra-ARMS marker may be more suitable for designing co-dominant type marker in autopolyploid plants. This preference arises because the tetra-ARMS marker targets a single SNP position, whereas the tri-ARMS marker necessitates two SNP positions with a cultivar-specific SNP in a cis relationship. If the cultivar-specific primers for a tri-ARMS marker are designed for two SNP positions with a cultivar-specific SNP in a trans relationship, such a marker cannot be applied to distinguish between homozygous and heterozygous genotypes during the breeding process (Supplemental Fig. 6). Based on the resequencing with short reads derived from autopolyploid, determining the cis/trans relationship among detected SNPs can be challenging. However, recent advances in long-read NGS platforms, such as NanoPore and PacBio, suggest that the cis/trans relationship for each allele at the detected SNP positions can be discerned by comparing the long reads (Yuan *et al.* 2021). If methods to identify two SNP positions with a trans relationship are established using long-read sequencing, the development of tri-ARMS markers for autopolyploid species will become feasible.

In DNAMarkMaker, the minimum coverage depth is a crucial consideration for developing DNA markers, especially when distinguishing heterozygous genotypes from homozygous genotypes at SNP positions in plants with heterozygous genome. Based on our simulation results presented in Supplemental Fig. 3, genomic regions with limited coverage depth make it challenging to distinguish between homozygous and heterozygous genotypes due to the fact that the 95% confidence interval for the SNP-index value ranges from ≥0 to ≤1.

For instance, in diploid plants, with a coverage depth of 5 or less, the 95% confidence interval spans a range from ≥0 to ≤1 for any given SNP position. This implies that homozygous SNP positions with SNP-index values of 0 or 1 cannot be reliably differentiated from heterozygous genotypes. Therefore, we recommend employing resequencing data with a coverage depth of at least 6 for diploid plants. At this depth, the 95% confidence interval for the SNP-index spans from >0.16 to <0.84, allowing for differentiation between heterozygous and homozygous genotypes.

For autotetraploid plants, the 95% confidence intervals for SNP-index values corresponding to simplex, duplex, and triplex types become discernible at coverage depths of 59 or greater. Additionally, when the developed DNA marker is applied to MAS in autotetraploid plants, selecting SNP positions having cultivar specific allele linked to the gene controlling target trait is necessary (Supplemental Fig. 7). Yamakawa *et al.* (2021) developed the strategy to identify the SNP positions conferring the allele linked to the gene controlling target trait in potato and sweet potato (*Ipomoea batatas*, an autohexaploid) by applying ‘polyploid QTL-seq’ analysis, an NGS-based bulked-segregant analysis in autopolyploid plants. Taken together, for developing DNA marker to apply to MAS in autopolyploid plants, enough coverage depth and selecting SNP position is required.

DNAMarkMaker is available both as a graphical user interface (GUI) and as a command user interface (CUI) tool. The CUI tool to researchers who are comfortable with command-line operations, such as the ability to create pipelines with other tools and improve the efficiency of parameter testing via parallel processing. This CUI version of DNAMarkMaker can be easily installed through the Bioconda platform (Grüning *et al.* 2018, https://github.com/SegawaTenta/DNAMarkMaker-CUI). The dual functionality of GUI and CUI in DNAMarkMaker extends its applicability not only to crop breeders but also to a wide range of biological researchers.

## Author Contribution Statement

TS developed DNAMarkMaker and set up the pipeline. SS contributed to develop the application’s GUI. MT and MH validated the developed DNA markers, while RK and HY provided support for PCR. HT designed the study, supervised it, and wrote the paper. All authors contributed to the manuscript writing.

## Supplementary Material

Supplemental Figures

Supplemental Tables
