# Supplementary material for: DNAMarkMaker: streamlining ARMS and CAPS marker development from resequencing data with NGS short reads
Source: Breed Sci. 2024 Feb 29;74(2):73–82. doi: 10.1270/jsbbs.23048 (PMC11442104; doi:10.1270/jsbbs.23048)
Supplement: Supplementary file 1 — Supplemental Figures [file 74_073_s1.pdf]

Segawa et al./Breeding Science
doi: 10.1270/jsbbs.23048

(A) tetra ARMS marker

Chrmosome	Position
chr11	24808079-24809366

Target SNP
24808426 (348)

Primers	Sequence	Length	Tm	GC%
Fw	AGTGGCATGCCCCGTGTTAA	20	62.4	55.0
Rv	TGTTGGAGGGAGGGAGCACT	20	62.6	60.0
I65_Fw	ATGCCGCAAAGACCGATCGA	20	62.5	55.0
IS68_Rv	CTAGTGCCGTCTGAGATCGC	20	60.3	60.0

- common Fw >>>>>>>>>
- common Rv <<<<<<<<<
- I65 specific Fw >>>>>>>>>
- IS68 specific Fw <<<<<<<<<

PCR product size
Fw & Rv : 1287
Fw & IS68_Rv : 367
I65_Fw & Rv : 959

PCR-amplified region

I65

```
AGTGGCATGCCCCGTGTTAACACTATGATAATATTTATATTAAGCAATCAGAAGATCATGTATAGTTTGGTAATTAAATATAAAATGTCATAAATTTTAA
>>>>>>>>>>>>>>>>>>>>
GAAATAAAAAATTCCAGACCAAGACATGTATAACACCGGTGTCAGATCCAAGTGATCTGTCCGACTCTTATCTACAGAATGAAACATTTTCATCTCCTTG

ATGTAATCCATCAAGTTATTTTTGTTGAGTACAACATGTTTAACAATCACAAGGAGGATCAGCTCAAGCAATCCAATCGAAACAACATCGATGATCATAA

GGTGTCATGATATAAAACTACGTGGCATATGCCGCAAAGACCGATTGACCATCTCAGACGGCACTAGAACAAGCTACAACCTCATTTGAACTATGGTTCT
                            >>>>>>>>>>>>>>>>>>>*<<<<<<<<<<<<<<<<<<<
TCATGGCCTGGAAGAGACCCTAATTGGCTAGTTGGGACGTGGAAATACCGAGTCACACCTGCAGTACTATATTTTGAAATTTGTTTTTAGATTTCATTTG

ATTTCTATAGCGAAATTTCTCTGTTTCCCCCCAAACTCTAAACTCCAATACCCTGAAGCATATAAATTGTCATCTGGCTCCAAATGTAATGATTGGCCAT

AAAAAAGCCATGTGTAAGAGCAAGTTTAATAGTATAGCCCACTACTAACTCCAATTCATTTATAGTCAATCTAATAGCTAATTCATACAATAGTTGCTTA

CTATACTATTAATATCTATGGTCCCCCCTGTCATACACACATTGCGTCTTGGAGTCCGTGCTGCAGCTGACTACAAATCTGTAGCCCGCTGCTCTTCTCT
                                                                     M
CTCTTCCGTTTCTCTCTTTAAAATATGTTTATAGCTGGCTTATAGCCTGCTATTGTACCTGCTCTAAATGCTACTTGTTTGCCTAGCCAGTTCTTTCCCA

TGGGTTCCTAACCCATTTCTATCGTTTTCCGCGTGCACGCTTTTTAAACTGCTAAATGGTACGTGTTCTGCGAAAAGTTTATATAGAAAAGTTGCTTTAA

ACAATCATATTCATTCTTTTTCAAGTTTTTTATAACTAATACTTAATAAATCATGTGTTAATATATCGCTTCGTTTTCCGTGCGGCGGGGAGGGGTTCCC

AACCCCTACAAAAGAACACAGATTTCTCCCATCCTTGAAGAGATACACACGTGTTTATATGCTTGAGATATGTTTTTTATAAACAGAGTTACTAAAACTA

CAGTTCAATAAATATATTGAAGAACTCTTATAATTCATAATGCTATAACACAAAACTACAGTTTCATAGTGCTCCCTCCCTCCAACA
                                                                   <<<<<<<<<<<<<<<<<<<<
```

IS68

```
AGTGGCATGCCCCGTGTTAACACTATGATAATATTTATATTAAGCAATCAGAAGATCATGTATAGTTTGGTAATTAAATATAAAATGTGATAAATTTTAA
>>>>>>>>>>>>>>>>>>>>
GAAATAAAAAATGCCAGACGTAGAGATGTATAACACCGGTGTCAGATCCAAGTTATCTTTCCTACTCTTATCTACAGAATGAAAAATTTTCATCTCCTTG

ATGTAATCCATCAATTTATTTTTGTTGAGTACCACATGTTTAACAATCACAAGCATCATCAGCTCAAGCAATCCAAGCGAAACCACATCGATGATCATAA

GGTGTCATGATATAAAGCTACGTGGCATATGCCGCAAAGACCGATTGGCCATCTCAGACGGCACTAGAACAAGCTACAACCTCATTTGAACTATGGTTCT
                            >>>>>>>>>>>>>>>>>>>*<<<<<<<<<<<<<<<<<<<
TCATGGCCTGGAAGAGACCCTAATTGGCTAGTTGGGACGTGCAAACAACGAGTCACACCTGCAGTACTATATTTTGAAATTTGTTTTTAGATTTCATTTG

ATTTCTATAGCGAAATTTCTCTGTTTCCCACCCAACTCTAAACGCCAATACCCTGAAGCATATAAATTGTCATCTGGCTCCAAATGTAATGATTGGCCAT

AAAAAAGCCATGTGTCAGAGCAAGTTTCATAGTATAGCCCTCCACTAACTTCAATTCATTTATAGTCAATCTAATAGCTCATTCATTCAATAGTTGCTTA

CTATACTATTAATATATATGGTCCCACCTGTCATACACACATTGCGTCTTGAAGCCCGTGCTTCAGCTCACTACAAATATGTAGCCCGCTGCTCTTCTCT
                                                                     M
CTCTTCCGTTTATCTCTTTAAAATATGTTTATAGCTGGCTTATAGCCTGCTATTGTACCTGCTCTAAATGCTACTTGTTTGCCTAGCCAGTTCTTTCCCA

TGGGTTCCTAACCCATTTCTATCGTTTTCCGCGCGCACGCTTTTCTTTCTGCTAAACGGTACGTGTTCTGCGAAAAGTTTATATAGAAAAGTTGCTTTAA

AAAATCATATTAATCCTTTTTCAAGTTTTTTATAACTAATACTTAATAAATCATATATTAATCTATCGCTTCGTTTTCCGTGCGGCGGGGAGGGGTTCCC

AACACCTACAAAAGAACACAGATCTCTCCCATCCTTGAAGAGATACACACCTGTTTATATGCTTGAGATATGTTTTTTATAAACAGAGTTACTAACACTA

CAGTTCAATAAATATATTGAAGAACTCTTATAATTCATAATGCTATAACACAAAACTACAGCTTCATAGTGCTCCCTCCCTCCAACA
                                                                   <<<<<<<<<<<<<<<<<<<<
```

Information for resequencing result

* : target SNP position on cultivar specific primers

(B)

CAPS marker

Chrmosome	Position
chr11	24647094-24647935

Target SNP

24647575 (482)

Restriction	Sequence	Sample
EcoRI	GAATTC	IS68

Primers	Sequence	Length	Tm	GC%
Fw	AGGTGTTTGGTGGCTGAGCA	20	62.2	55.0
Rv	TGCTGCTTGCTAAGGCCTGG	20	63.1	60.0

Fw and Rv primers

PCR product size

Fw & Rv : 841

PCR-amplified region

I65

```
AGGTGTTTGGTGGCTGAGCACATGTTGGAATCAGAACATGTTTGAGCTGAATTTGTGGATGCATGAACAGGTGTTTGGAATTCTCAAAAAAAAAAAAAAG
>>>>>>>>>>>>>>>>>>>>
GAGCAGGTGTTTGTGGACAATTTGCAGCGTTGTTGACTGTAATCAGACGGTGGCTATGTTGACTTCAACACAGTATTGGTTGAGATTACCAAAACAATCA
            +0
TCGGAGCATGGGGATTTTCTGAATGTTTTGGGGATGATCCTTTGAAACTGAAAGTAGTGAGAATGCTTCTTGCTTCAGAGGCAAAAGTGAAGCTGCCCAT
             M
GTGGGCGCCACCTGATTAGCAACCCTAAATCATCAATAAATCAATTAATTAGTAATTCAAAGTACTAAACACGCACGCATTGCCGTGTTGTACACCTATC

TTGAGATTAGAGGAGCCAAGCATCATCTTCCATCGGGCGAAGAAAGAATCTGCACCAAGACGGAGCCAGCAACTGAGAATTTAGGCCCCGTTTAGTTCCA

AAAATTTTTTTCCCCAAAAACATCATATCGAATATTTAGACACATACATAAAATATTAAATATAAATAAAAAAAACTAATTACACAGTTTGCATAAAAAT
                                                                -LLLLL000
CACGAGACAAATCTTTTGAGCCTAATTAGTTCAGGATTAGCCATAAGTGCTACAATGACCCACATGTGTTAATAACGTATTAATTATGCTCAAAAAATTC

GTCTCGCGGTTTCTAGGTGAGTTATGAAATTAGTTTTTTATTCATGTCAAAACCTCTTCTGAATAAACATTCAATTAAACGTCCGATGTGACACTCAGTA

ATTTTCTTTTCACGAACTAAACCAGGCCTTAGCAAGCAGCA
                    <<<<<<<<<<<<<<<<<<<<
```

IS68

```
AGGTGTTTGGTGGCTGAGCACATGTTGGAATCAGCACATGTTTGAGCTGAATTTGTGGATGCATGAACAGGTGTTTGGAATTATCAAAAAAAAAAAAAAG
>>>>>>>>>>>>>>>>>>>>
GAGCAGGTGTTTGTGGATAAGTTGCAACGTTGTTGACTGTAATCAGACGGTGGCTATGTTGACTTCAACACAGTATTGGTTGAGATTACCAAAACAATCA

TCGGAGCATGGGGATTTTATGAATGTTTTGGGGATGATCCTTTAAAACTGAAAGTAGTGAGAATGCTTCTTGCTTCAGAGGCAAAAGTGAAGCTGCCCAT
             M
GTGGGCGCCACCTGATTAGCAACCCTAAATCATCAATCAATCAATTAATTAGTAATTCAAAGTACTAAACACGCACGCATTGCCGTGTTGTACACCTATC

TTGAGATTAGAGAAGCCAAGCATCATCTTCCATCGGGCGAAGAAAGAATCTGCACCAAGACGGAGTCAGTAACTGAGAATTCAGGCCCCGTTTAGTTCCA

AAAATATTTTTCCCTAAAAACATCATATCGACTATTTAGACACATACATAAAGTATTAAATATAAATAAAAAAAACTAATTGCACAGTTTGCATAAAAAT

CACGAGACAAATCTTTTGAGCCTAATTAGTCCATGATTAACCATAAGTGCTACAGTGACCCACATGTGTTAATAACGTATTAATTATGCTCAAAAAATTC

GTCTCGCGCTTTCTAGGTGAGTTATGAAATTAGTTTTTTATTCGTGTCAAAACCTCTTCTTAATGAACATTTAGTTAAACGTCCGATGTGACACTCAATA

ATTTTCTTTTCACGAACTAAACCAGGCCTTAGCAAGCAGCA
                    <<<<<<<<<<<<<<<<<<<<
```

Information for resequencing result

GAATTC blue : restriction enzyme specific region

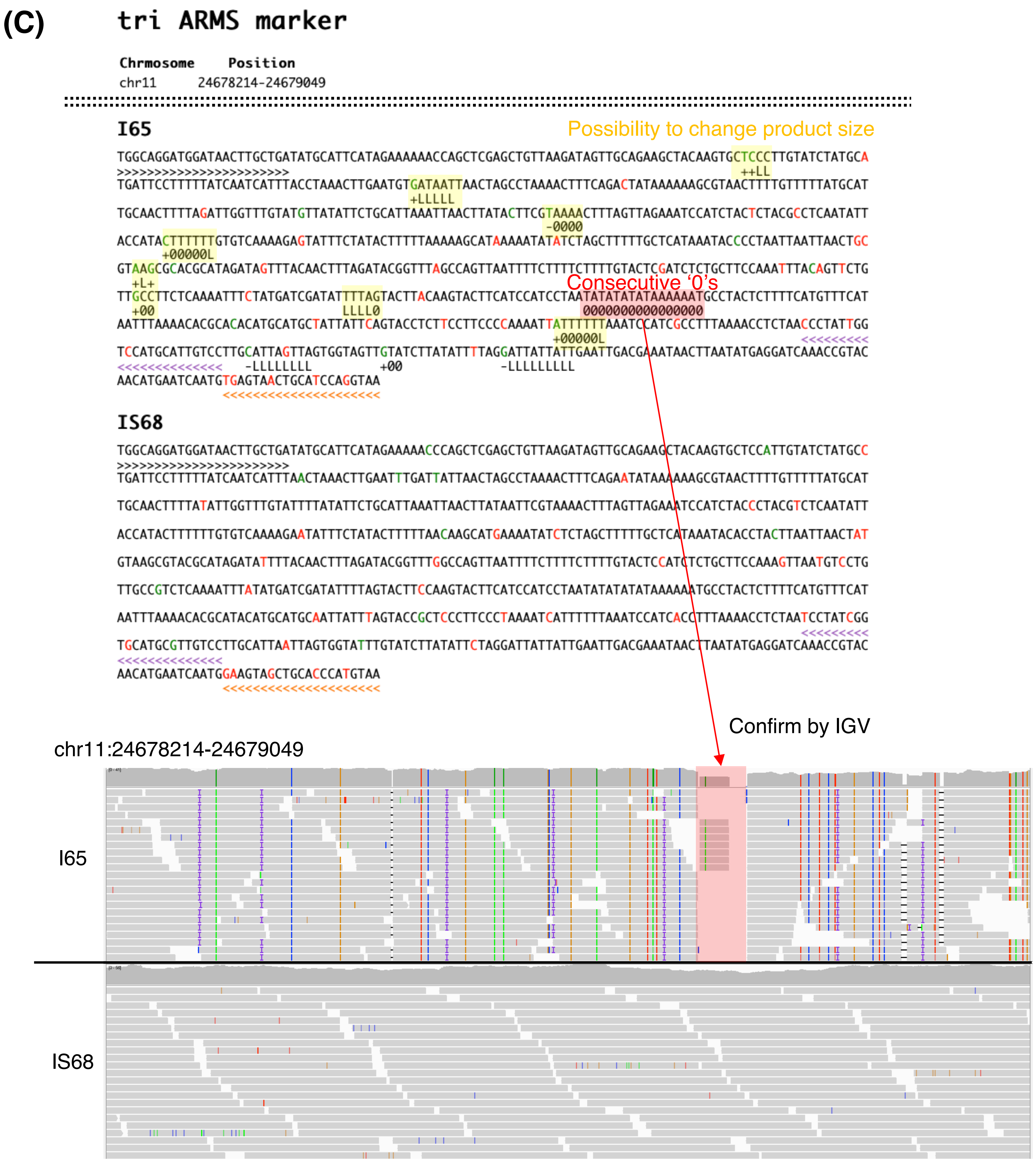

Supplemental Fig. 1.
The interpreted HTML file output from DNAMarkMaker, showcasing the design of tetra-ARMS (A) and CAPS (B) markers. (C) Points to note when selecting markers.

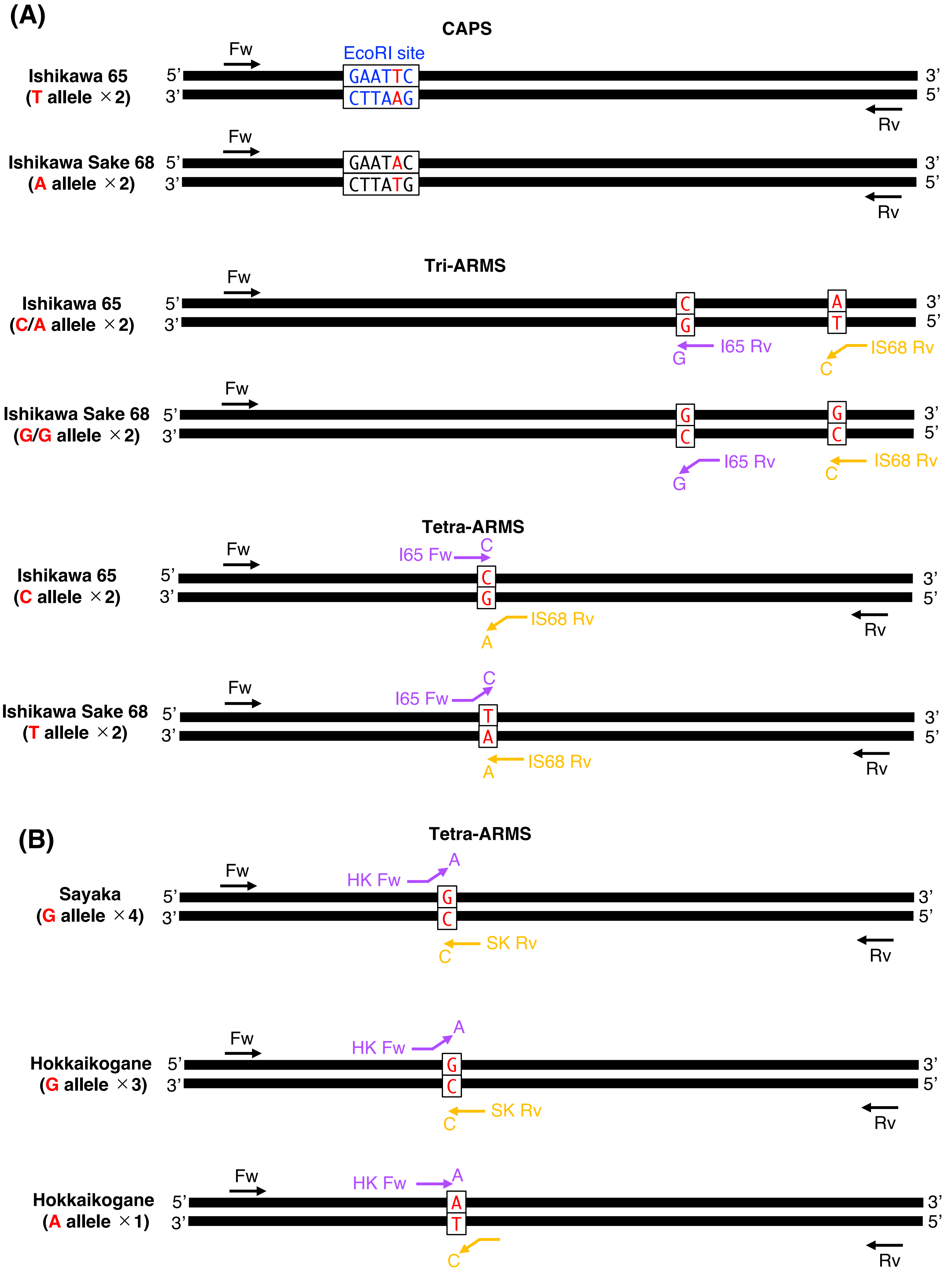

(A)
CAPS
Fw
EcoRI site
Ishikawa 65
(T allele ×2)
5’
3’
GAATTC
CTTAAG
3’
5’
Rv
Fw
Ishikawa Sake 68
(A allele ×2)
5’
3’
GAATAC
CTTATG
3’
5’
Rv
Tri-ARMS
Fw
Ishikawa 65
(C/A allele ×2)
5’
3’
C
G
A
T
3’
5’
G
I65 Rv
C
IS68 Rv
Fw
Ishikawa Sake 68
(G/G allele ×2)
5’
3’
G
C
G
C
3’
5’
G
I65 Rv
C
IS68 Rv
Tetra-ARMS
Fw
I65 Fw
C
Ishikawa 65
(C allele ×2)
5’
3’
C
G
3’
5’
IS68 Rv
A
Rv
Fw
I65 Fw
C
Ishikawa Sake 68
(T allele ×2)
5’
3’
T
A
3’
5’
IS68 Rv
A
Rv
(B)
Tetra-ARMS
Fw
HK Fw
A
Sayaka
(G allele ×4)
5’
3’
G
C
3’
5’
SK Rv
C
Rv
Fw
HK Fw
A
Hokkaikogane
(G allele ×3)
5’
3’
G
C
3’
5’
SK Rv
C
Rv
Fw
HK Fw
A
Hokkaikogane
(A allele ×1)
5’
3’
A
T
3’
5’
C
Rv

Continued...

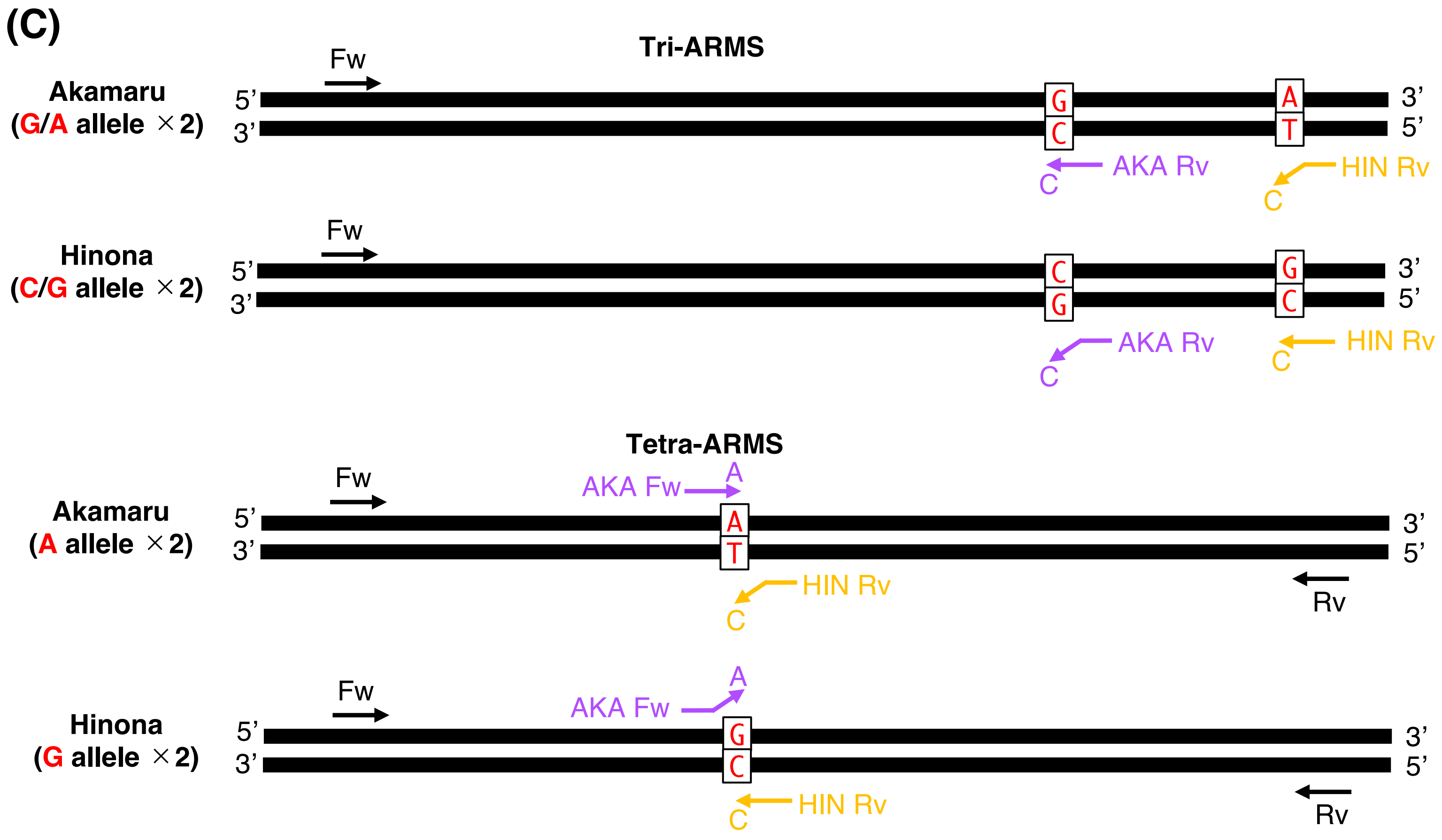

Supplemental Fig. 2: Schematic diagram of primer sets for validating the function.
(**A**) Marker sets for rice in Fig. 4. (**B-C**) Marker sets in Fig. 5: potato (B) and turnip (C). Red and blue letters indicate SNP sites and restriction enzyme site, respectively. Both purple and orange primers represent cultivar-specific primers.

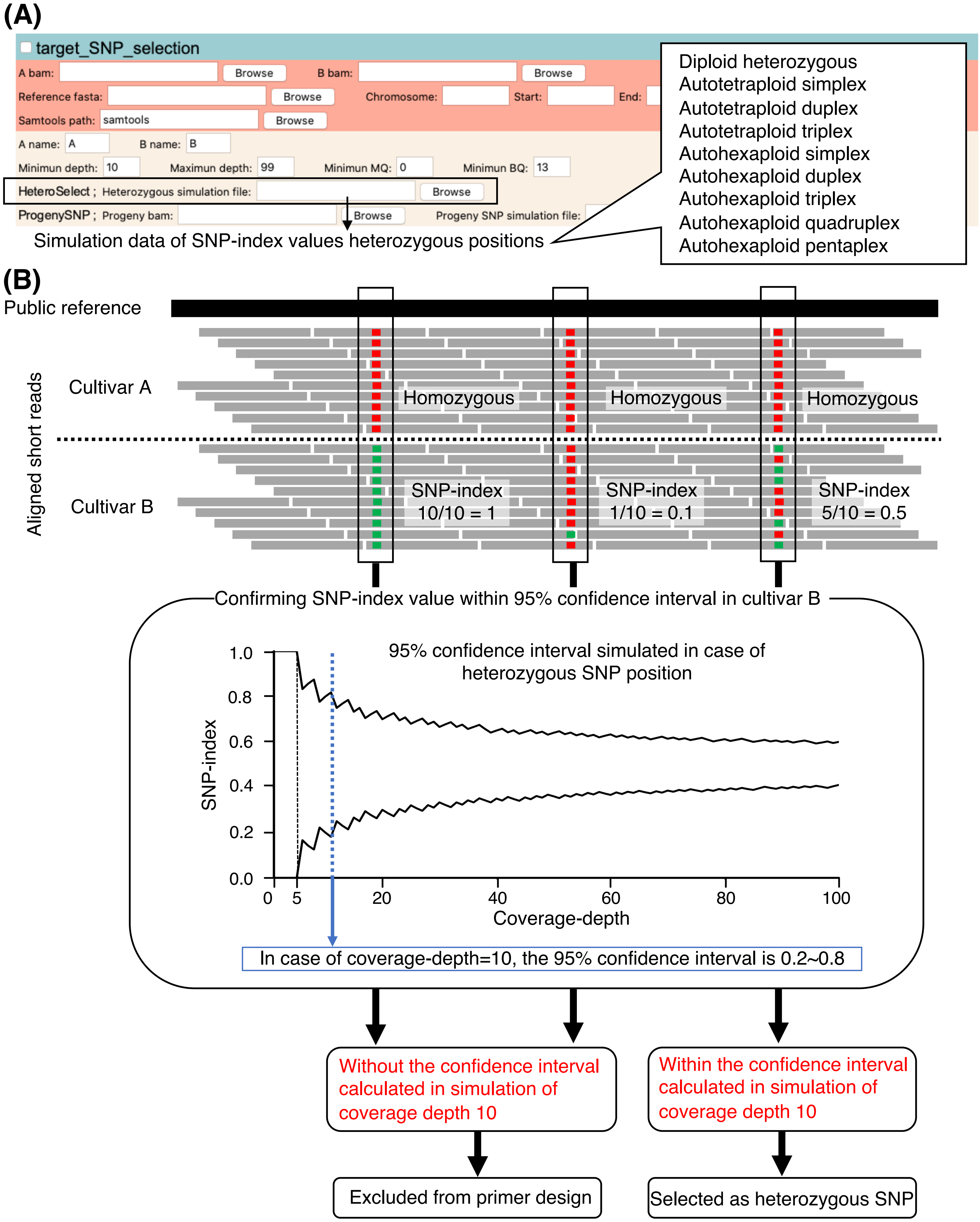
(A)
target_SNP_selection
A bam: Browse B bam: Browse
Reference fasta: Browse Chromosome: Start: End:
Samtools path: samtools Browse
A name: A B name: B
Minimun depth: 10 Maximun depth: 99 Minimun MQ: 0 Minimun BQ: 13
HeteroSelect ; Heterozygous simulation file: Browse
ProgenySNP ; Progeny bam: Browse Progeny SNP simulation file:
Simulation data of SNP-index values heterozygous positions
Diploid heterozygous
Autotetraploid simplex
Autotetraploid duplex
Autotetraploid triplex
Autohexaploid simplex
Autohexaploid duplex
Autohexaploid triplex
Autohexaploid quadruplex
Autohexaploid pentaplex
(B)
Public reference
Aligned short reads
Cultivar A
Homozygous
Homozygous
Homozygous
Cultivar B
SNP-index 10/10 = 1
SNP-index 1/10 = 0.1
SNP-index 5/10 = 0.5
Confirming SNP-index value within 95% confidence interval in cultivar B
95% confidence interval simulated in case of heterozygous SNP position
SNP-index
1.0
0.8
0.6
0.4
0.2
0.0
0
5
20
40
60
80
100
Coverage-depth
In case of coverage-depth=10, the 95% confidence interval is 0.2~0.8
Without the confidence interval calculated in simulation of coverage depth 10
Within the confidence interval calculated in simulation of coverage depth 10
Excluded from primer design
Selected as heterozygous SNP

(C)

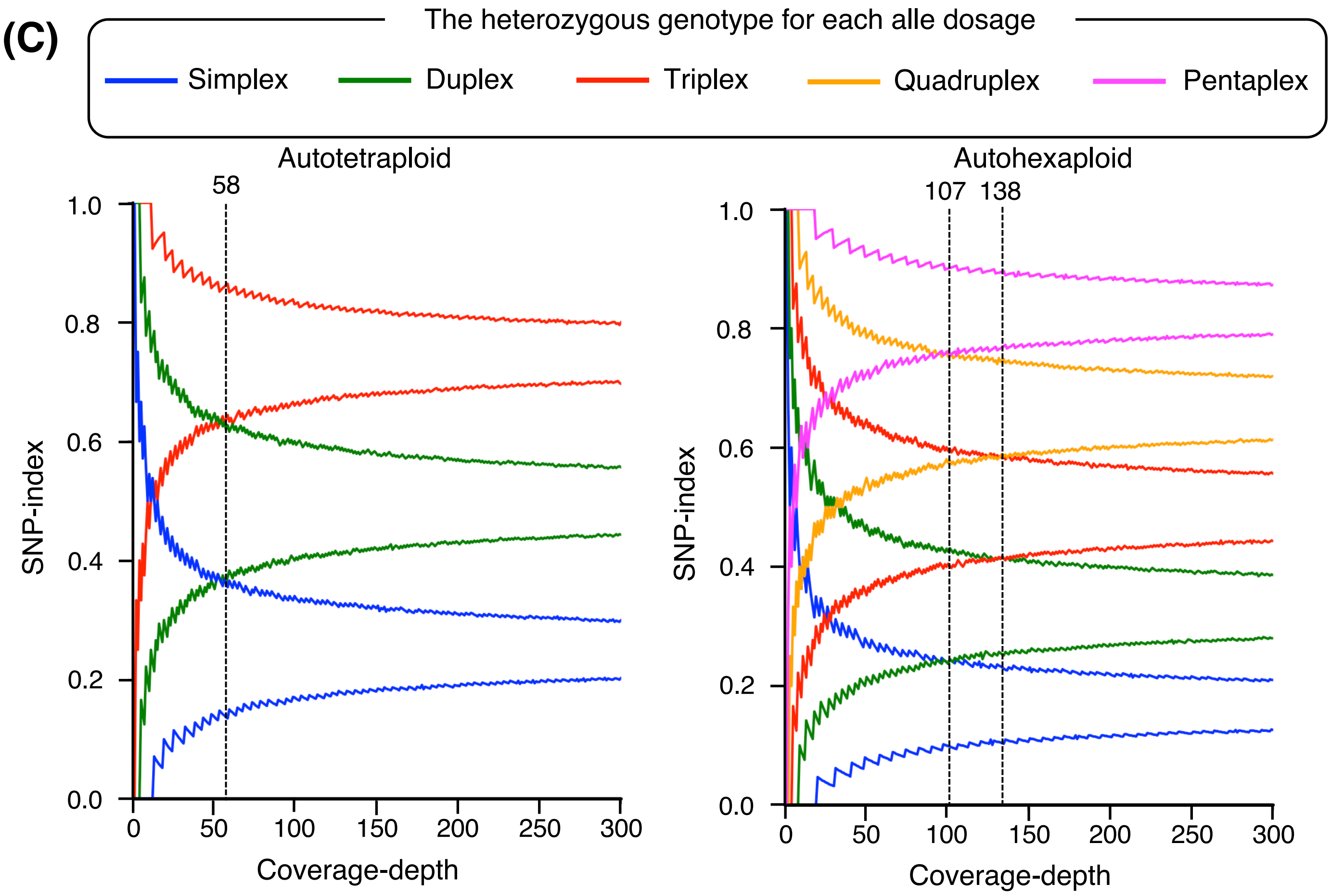

Supplemental Fig. 3: Utilizing the "HeteroSelect" option in "target_SNP_selection".
(**A**) The DNAMarkMaker tool's input screen when using the "HeteroSelect" option. The nine simulation data sets utilized in the heterozygous simulation column of "HeteroSelect" are accessible in GitHub (https://github.com/SegawaTenta/DNAMarkMaker_manual). (**B**) The flowchart of the selection process of heterozygous SNP positions in diploid plants using the "HeteroSelect" option in "target_SNP_selection". The simulation process used was the same as that described in Supplemental Fig. 1 in our previous study (Itoh *et al*. 2016, *Theor Appl Genet*, 132, 2913-2925). (**C**) The 95% confidence interval is calculated in the simulation of the SNP index value for each coverage depth in autopolyploid. Each simulated genotype is represented in a different color.

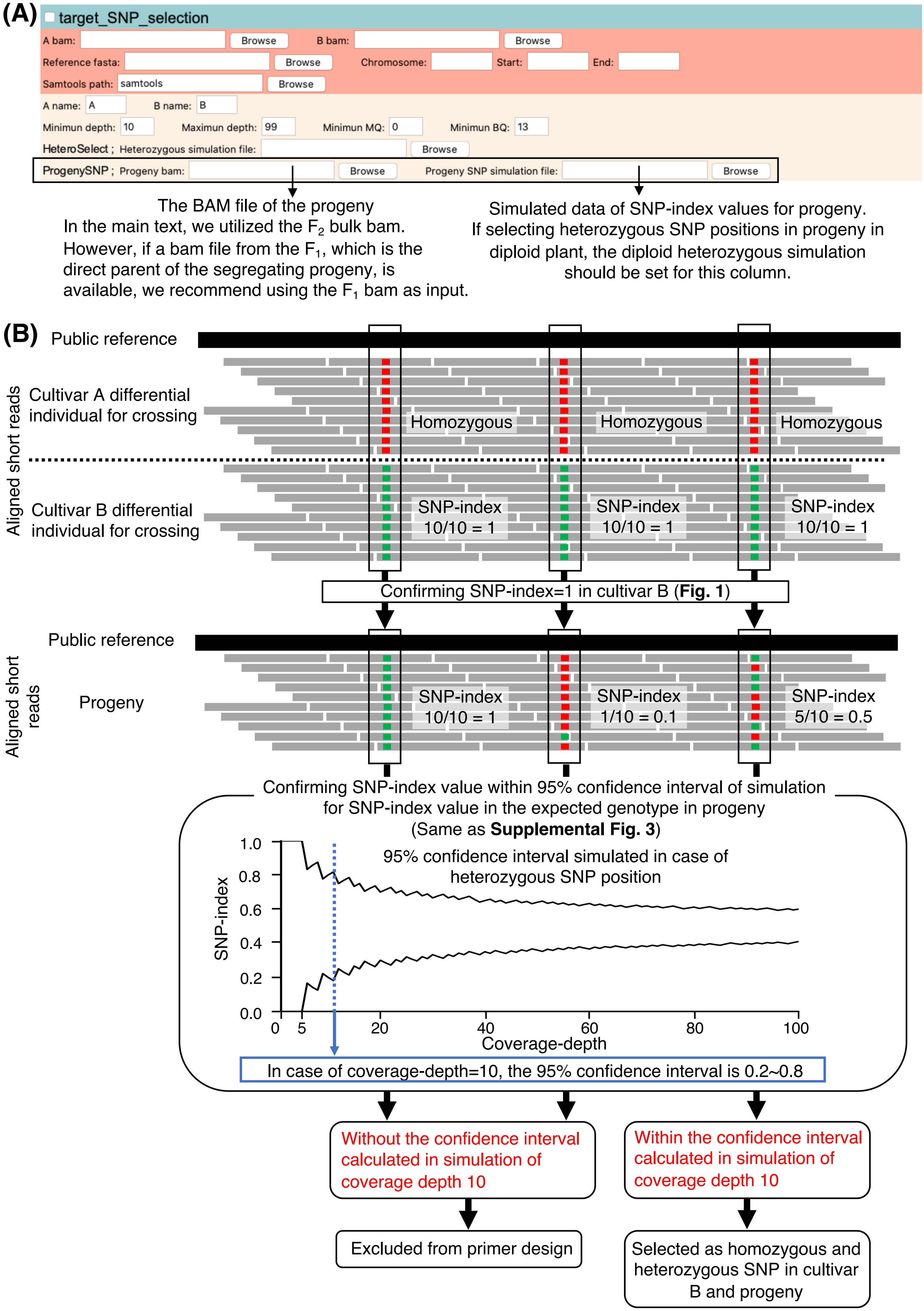
(A)
target_SNP_selection
A bam:
Browse
B bam:
Browse
Reference fasta:
Browse
Chromosome:
Start:
End:
Samtools path:
samtools
Browse
A name:
A
B name:
B
Minimun depth:
10
Maximun depth:
99
Minimun MQ:
0
Minimun BQ:
13
HeteroSelect ; Heterozygous simulation file:
Browse
ProgenySNP ; Progeny bam:
Browse
Progeny SNP simulation file:
Browse
The BAM file of the progeny
In the main text, we utilized the F_2 bulk bam. However, if a bam file from the F_1, which is the direct parent of the segregating progeny, is available, we recommend using the F_1 bam as input.
Simulated data of SNP-index values for progeny. If selecting heterozygous SNP positions in progeny in diploid plant, the diploid heterozygous simulation should be set for this column.
(B)
Public reference
Aligned short reads
Cultivar A differential individual for crossing
Homozygous
Homozygous
Homozygous
Cultivar B differential individual for crossing
SNP-index 10/10 = 1
SNP-index 10/10 = 1
SNP-index 10/10 = 1
Confirming SNP-index=1 in cultivar B (Fig. 1)
Public reference
Aligned short reads
Progeny
SNP-index 10/10 = 1
SNP-index 1/10 = 0.1
SNP-index 5/10 = 0.5
Confirming SNP-index value within 95% confidence interval of simulation for SNP-index value in the expected genotype in progeny (Same as Supplemental Fig. 3)
95% confidence interval simulated in case of heterozygous SNP position
SNP-index
1.0
0.8
0.6
0.4
0.2
0.0
0
5
20
40
60
80
100
Coverage-depth
In case of coverage-depth=10, the 95% confidence interval is 0.2~0.8
Without the confidence interval calculated in simulation of coverage depth 10
Within the confidence interval calculated in simulation of coverage depth 10
Excluded from primer design
Selected as homozygous and heterozygous SNP in cultivar B and progeny

Supplemental Fig. 4: Utilizing the "ProgenySNP" option in "target_SNP_selection".

(**A**) The DNAMarkMaker tool's input screen when using the "ProgenySNP" option. The simulation data sets are accessible in GitHub (https://github.com/SegawaTenta/DNAMarkMaker_manual). (**B**) The flowchart represents the process of selecting SNP positions in diploid plants using the "ProgenySNP" option in "target_SNP_selection". "ProgenySNP" allows the selection of SNPs from the positions detected in the resequencing of parent lines, by comparing positions where the genotype defined by resequencing in the progeny corresponds to the expected genotype. In the main text's example, "ProgenySNP" selected positions from the homozygous SNP positions between turnip cultivars 'Akamaru' and 'Hinona' based on the positions that exhibited a heterozygous genotype in the F_2 bulked resequencing data.

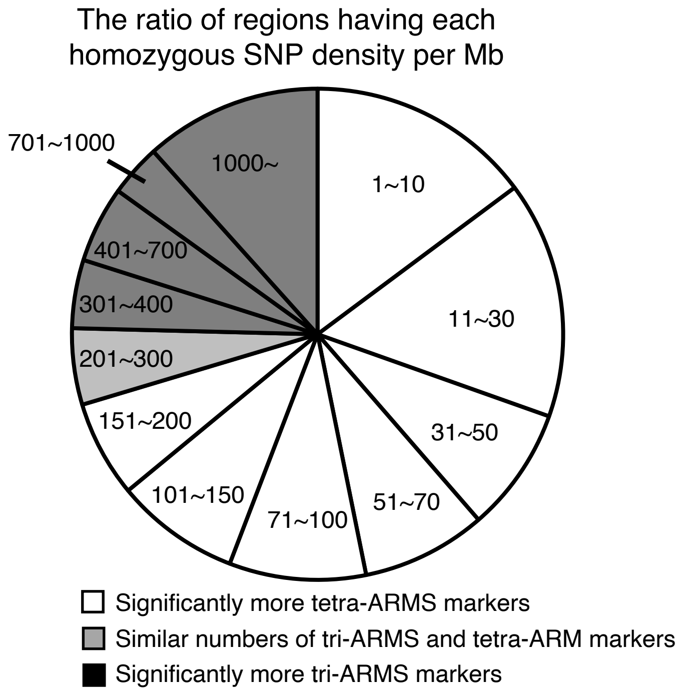

Supplemental Fig. 5: Comparison between the number of tri-ARMS versus tetra-ARMS in each SNP density in rice sample used from Fig. 4.

The each range on pie chart shows the SNP density per Mb. A comparison of the number of tri-ARMS versus tetra-ARMS markers for each SNP density was conducted in the 10 randomly selected 1 Mb regions. A significant difference was identified with a *p*-value of < 0.05, as determined by a two-sample *t*-test.

Tri-ARMS marker designed at dual simplex SNPs with a trans relation in cultivar-B

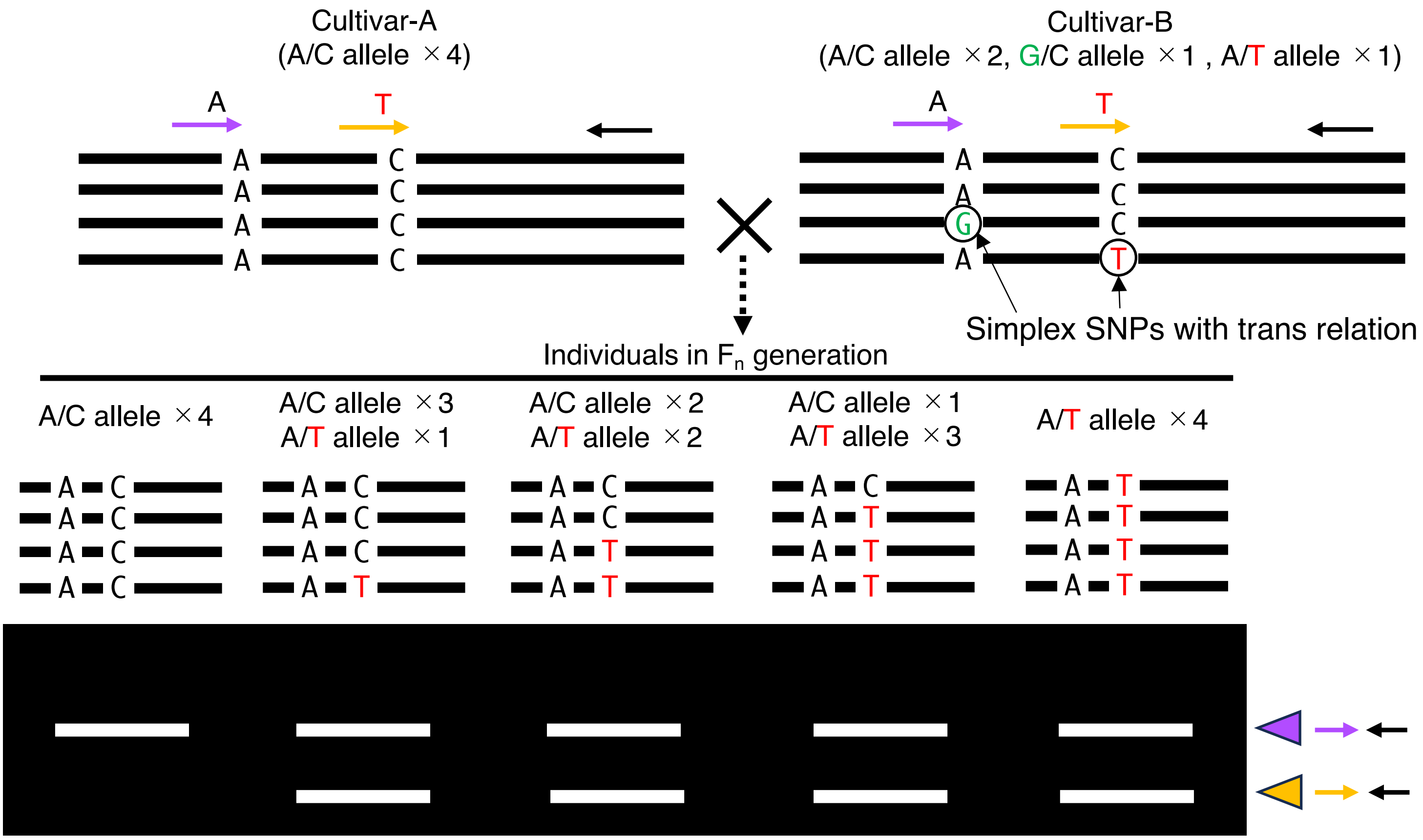

Tri-ARMS marker designed at dual simplex SNP positions with a cis relation in cultivar-B

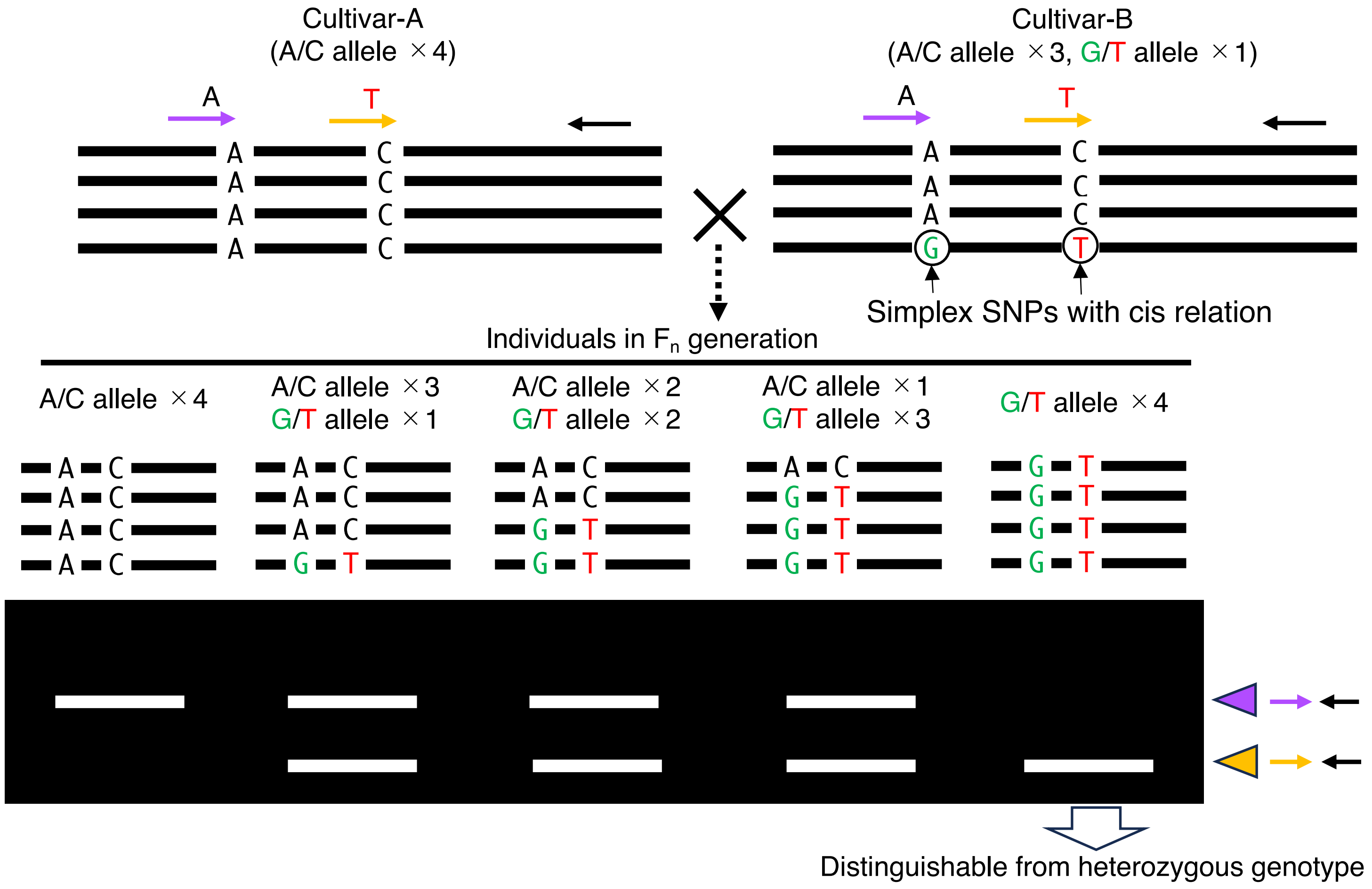

Supplemental Fig. 6. Continued...

Tetra-ARMS marker

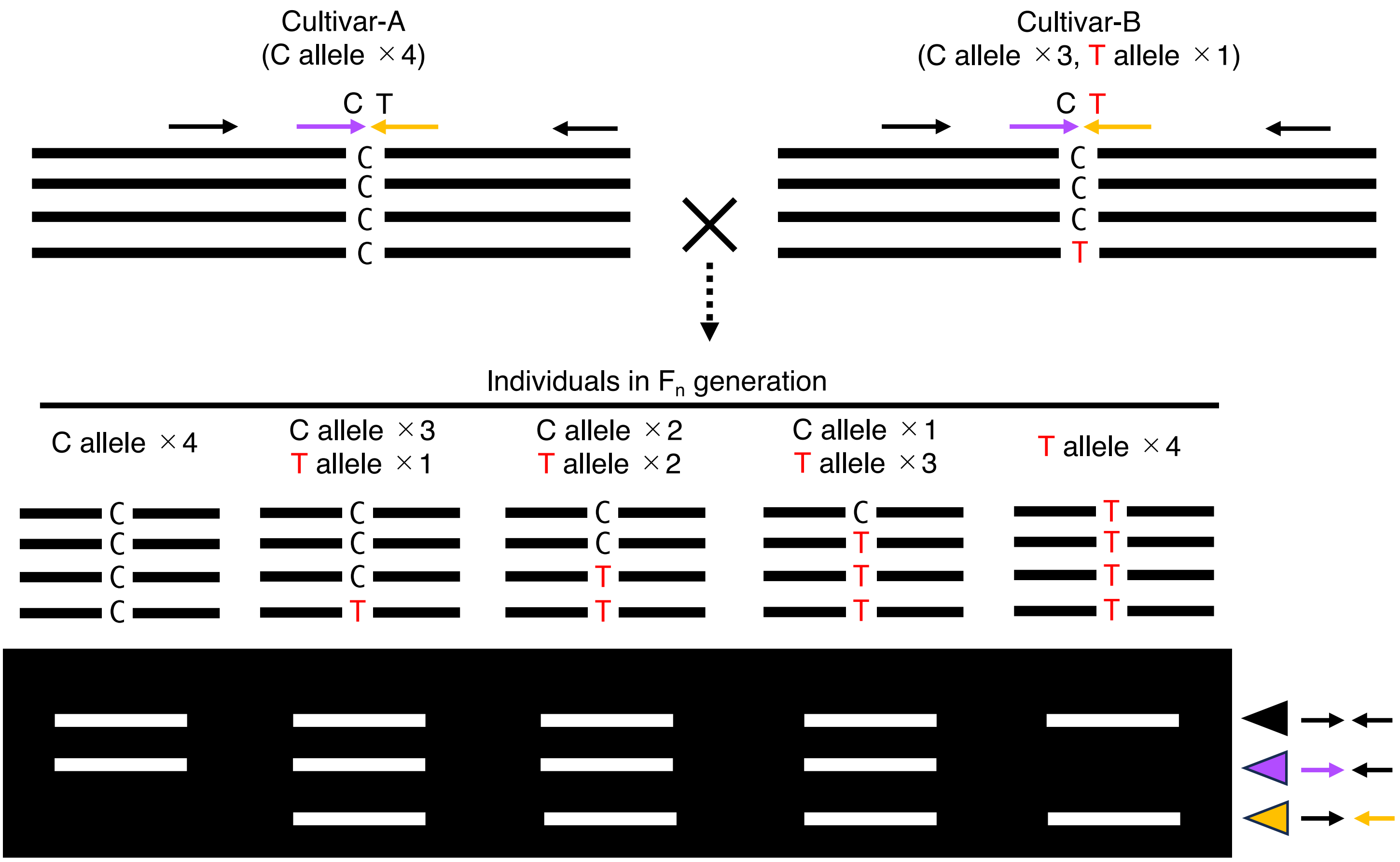

Supplemental Fig. 6. Electrophoresis images of the PCR products obtained using each type of ARMS marker developed by DNAMarkMaker.
The tri-ARMS marker, designed at dual SNP positions for a cultivar-B specific allele with a trans relation, cannot be employed as a co-dominant type marker.

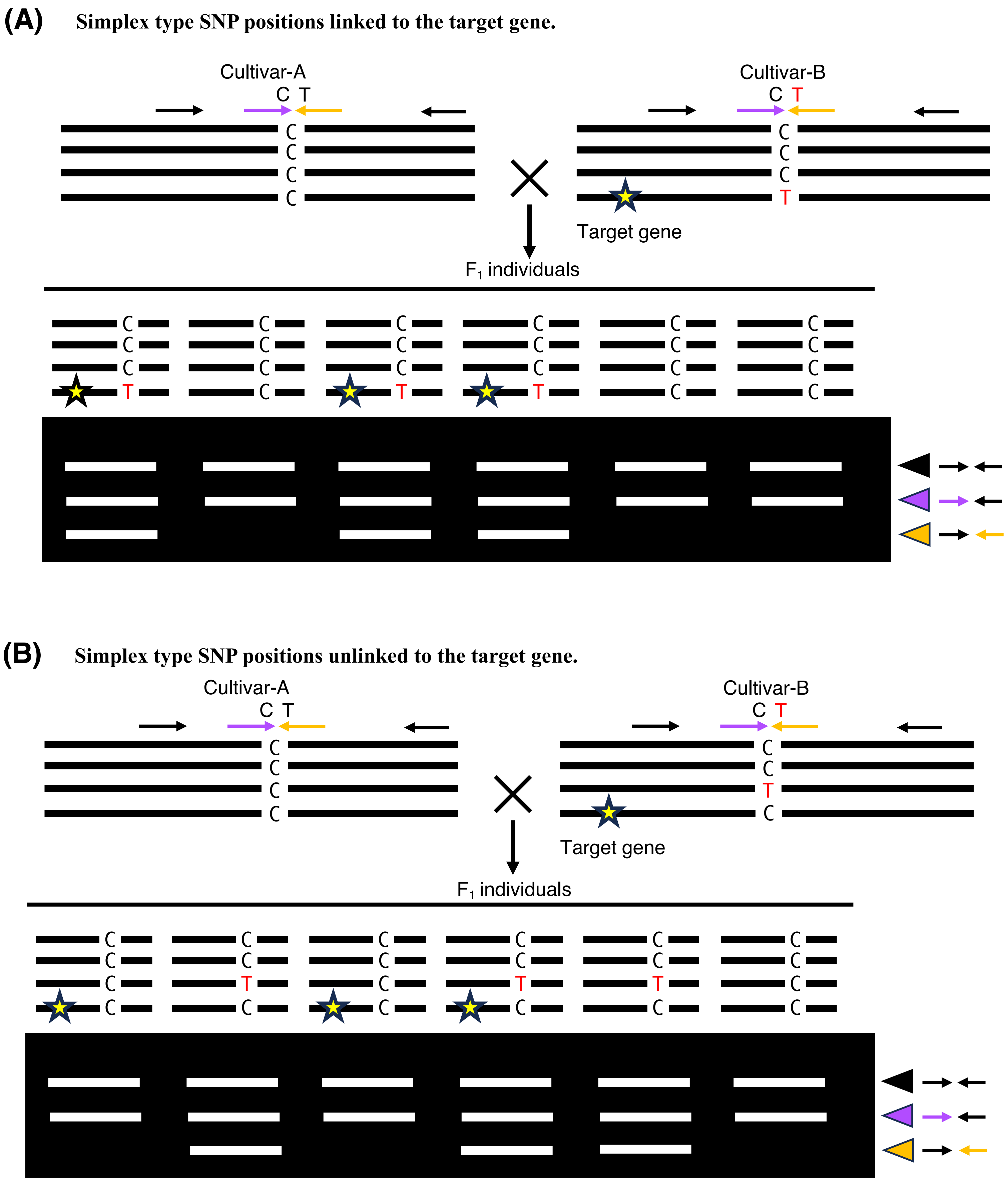

Supplemental Fig. 7. Electrophoresis images showcasing the PCR products derived using tetra-ARMS markers, designed at simplex type SNP positions that are either linked (A) or unlinked (B) to the target gene.
