# Supplementary material for: DNAMarkMaker: streamlining ARMS and CAPS marker development from resequencing data with NGS short reads
Source: Breed Sci. 2024 Feb 29;74(2):73–82. doi: 10.1270/jsbbs.23048 (PMC11442104; doi:10.1270/jsbbs.23048)
Supplement: Supplementary file 2 — Supplemental Tables [file 74_073_s2.pdf]

doi: 10.1270/jsbbs.23048

Supplemental Table 1. Summary of Illumina pair-end short reads.

Crops	Cultivars	Accession number
Rice	Ishikawa 65	DRR493709
	Ishikawa Sake 68	DRR493710
Potato	Sayaka	DRR255454
	Hokkaikogane	DRR255455
Turnip	Akamaru	DRR218913
		DRR218914
	Hinona	DRR218919
		DRR218920
	All F_2 bulk (A×H)	DRR218903
		DRR218904

Supplemental Table 2. Summary of option in “target_SNP_selection”.

	**Rice**	**Poteto**	**Turnip**
**A bam**	I65	Sayaka	Akamatu
**B bam**	IS68	Hokkaikogane	Hinona
**C bam**	-	-	F_2 random bulk
**Reference fasta**	IRGSP-1.0	DM 1-3 516 R44	Chiifu V4.0
**Minimum depth**	10	59	6
**Maximum depth**	99	300	99
**HeteroSelect simulation file**	-	Simplex for autotetraploid	-
**ProgenySNP simulation file**	-	-	Heterozygous for diploid
